# Management of early-onset scoliosis: modern Luque trolley technique led to fewer reoperations within 3 years than other growth-friendly techniques, a prospective cohort study with matched historical controls

**DOI:** 10.1007/s43390-025-01102-2

**Published:** 2025-05-28

**Authors:** Romain Dayer, Michael Grevitt, Lee Breakwell, WaiWeng Yoon, Maio Chen, Tiara Ratz, Tina Szocik, Yaner Y. Zhu, Pediatric Spine Study Group (PSSG), Jean Ouellet

**Affiliations:** 1 Pediatric Orthopaedics Unit, Geneva University Hospitals, Geneva, Switzerland; 2 Centre for Spinal Studies and Surgery, Queens Medical Centre Campus, Nottingham University Hospitals NHS Trust, Nottingham, UK; 3 Sheffield Children’s Hospital, Western Bank, Sheffield, UK; 4 AO Innovation Translation Center, Clinical Operations, AO Foundation, Davos, Switzerland; 5 Pediatric Spine Foundation, Valley Forge, PA USA; 6 AO Innovation Translation Center, Medical Scientific Affairs, AO Foundation, Davos, Switzerland; 7 Department of Paediatric Surgery, McGill University, Montreal, QC Canada

**Keywords:** Early-onset scoliosis, Pediatric spinal deformity, Modern Luque technique, Growing rods, Implant failure

## Abstract

**Purpose:**

Management of early-onset scoliosis (EOS) remains challenging with high reoperation rates. The modern Luque trolley technique (MLT) was developed to reduce open lengthening and complications. This study aimed to compare the reoperation rates between the MLT and other growth-friendly surgical techniques.

**Methods:**

Prospective EOS patients were recruited and treated with MLT; matched historical controls were selected from the Pediatric Spine Study Group (PSSG) database. The primary objective was to test if within 3 years of surgery MLT patients would have fewer reoperations. Secondary outcomes were growth, curve correction, and quality of life using the 24-item early-onset scoliosis questionnaire (EOSQ-24). Safety analysis was performed for the MLT patients.

**Results:**

MLT (N = 18) and control patients (N = 43) had similar baseline age, body measurements, etiology, Cobb angle, and spinal length. Within 3 years of surgery, 1/18 MLT patients required a reoperation compared with 30/43 controls, conditional Poisson regression rate ratio = 0.02 (95% CI 0; 0.12) (P < 0.001). The median time to first reoperation was MLT, 5.4 years and control, 0.8 years. The MLT achieved the same curve correction as the controls at 3 years. The total spinal growth (T1–S1) was similar between the groups, although the thoracic spinal growth (T1–T12) was less in the MLT group. No difference was observed in standing heights and EOSQ-24 scores. Within 3 years, 2 MLT patients had recurrence of deformity (risk = 11.1%, 95% CI 1.4; 34.7) and 1 had implant loosening (risk = 5.6%, 95% CI 0.1; 27.3).

**Conclusion:**

MLT patients had fewer reoperations within 3 years than control patients and a low risk of implant failure.

**Levels of evidence:**

Level II.

**Trial registration number:**

NCT01672749. *Date of registration*: 2012-08-24.

## Introduction

The management of early-onset scoliosis (EOS) is challenging through the need to control spinal deformity while allowing for normal spinal growth and lung development. Growing rods have been widely used in the past 2–3 decades but high complication rates, repeated lengthening surgeries, and autofusion remain serious concerns [1, 2].

The original Luque trolley offered a novel approach to segmental fixation without fusion, whilst allowing self-guided growth [3]. Despite the initial promise, the technique led to significant complications and limited growth and was abandoned for other growth-friendly techniques such as the dual growing rods (DGR), rib-based growing rods (RBGR), and magnetically controlled growing rods (MCGR) [1, 4]. In the past decade, MCGR has become the predominant technique, used in 83% of index operations in EOS [5]. However, with longer follow-up, high complication and revision rates were also observed [6, 7].

**Fig. 1:**
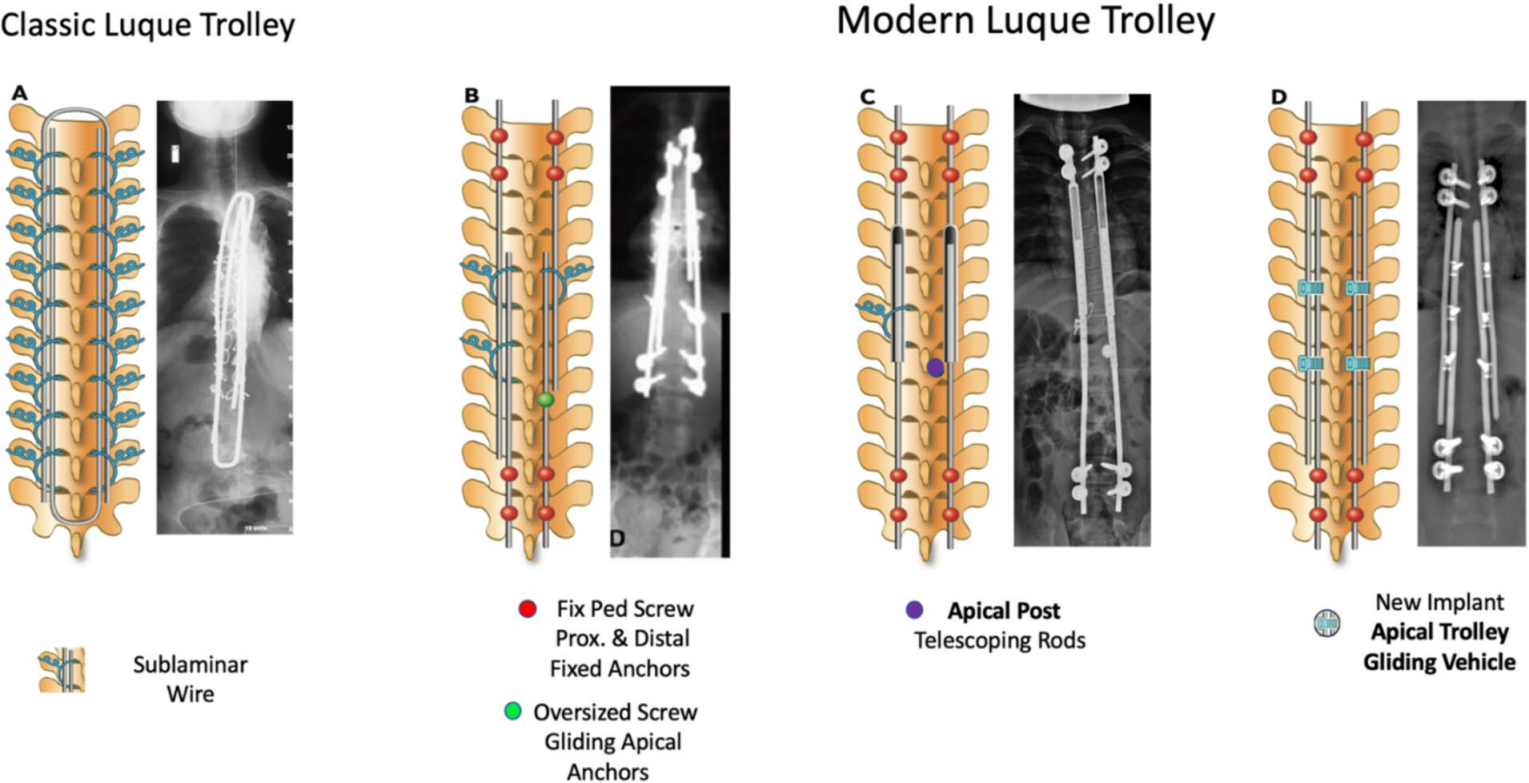
Types of constructs adopting the Luque Trolley concept of self-growth guidance. The classic Luque trolley technique with sublaminar wires (**A**) or a combination of sublaminar wire, fixed pedicle screw proximal and distal fixed anchors, and screw gliding apical anchors (**B**). The modern Luque trolley technique progressed from telescoping rods (**C**) to the novel implant used in the current study consisting of fixed distal and proximal anchors and the apical trolley gliding vehicles (**D**)

**Fig. 2:**
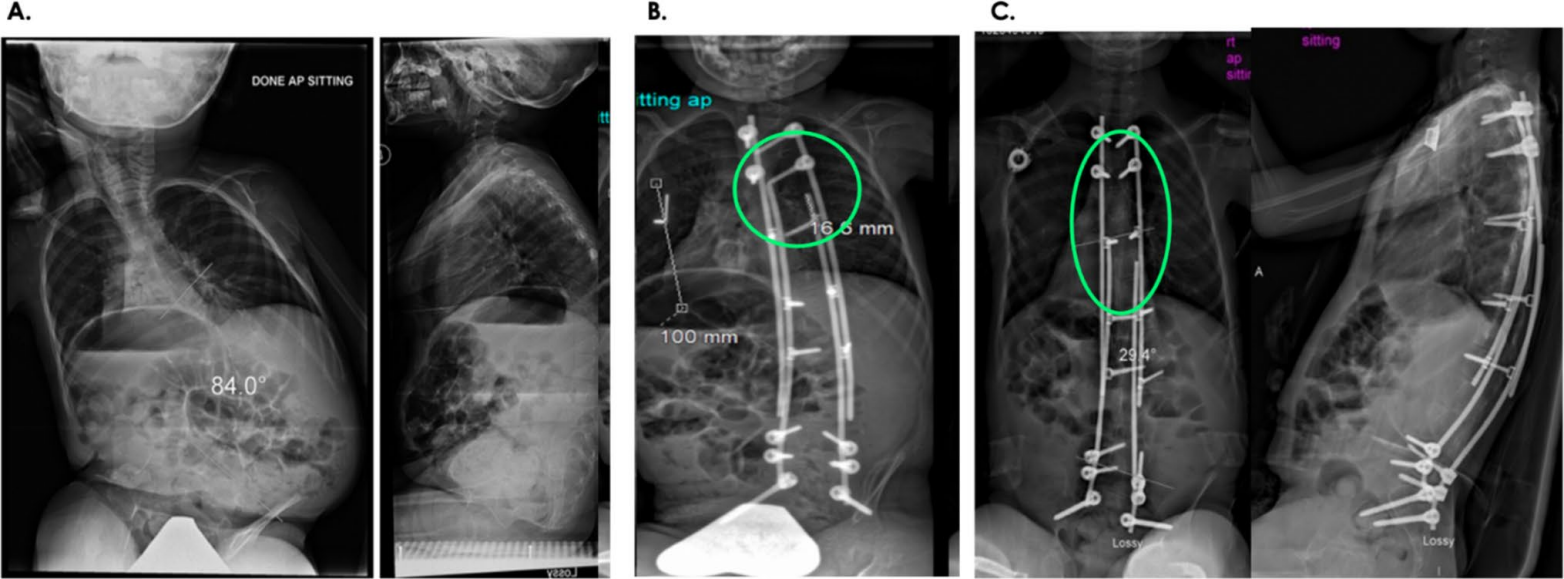
Clinical example of the modern Luque trolley technique. **A** The modern Luque trolley technique was performed on a 6-year-old with preoperative low tone severe scoliosis of 84°. **B** Immediately post operation, the deformity was corrected to 28°. **C** At 5 years follow-up, the trolley gliding vehicles had a self-guided growth of 5.5 cm (growth within the green circles from **B** to **C**). The patient did not require any revision surgery

The modern Luque trolley technique (MLT) retains the initial concept of self-guided growth but with new apical gliding spinal anchors and minimal-invasive approach (see Fig. 1) [8]. The current study examined the performance of the MLT with the newly designed gliding implant (see Fig. 2) and compared its reoperation rate with other growth-friendly techniques.

## Methods

This is a prospective, multicenter cohort study with matched historical control. The MLT group included patients treated with the MLT; the control group comprised matched patients treated with DGR, RBGR, or MCGR selected from the Pediatric Spine Study Group (PSSG) database (https://pediatricspinefoundation.org/pediatricspinestudy.aspx). A 1:1 match of MLT patients with patients treated with each of the three growth-sparing techniques was intended.

### Objectives

The primary objective of the study was to compare the 3-year reoperation (of the spine) rate; we hypothesized that the MLT group would have fewer reoperations.

Secondary objectives included comparison of growth, deformity, and quality of life. Adverse events in the MLT group were also recorded.

### Study design

Patients in the MLT group were prospectively recruited from three sites in Switzerland and the United Kingdom. Demographics, physical measurements, etiology of scoliosis, spinal length, and deformity (Cobb angle) were recorded at baseline preoperatively; surgical details were collected intraoperatively and radiographs taken as customary at participating sites. Patients were prospectively followed up at 6 weeks, 6-monthly up to 3 years, and then annually up to 10 years.

### Inclusion and exclusion criteria

The inclusion criteria of the MLT group were patients 5–10 years old with pre-peak growth velocity, bone age < 10 years, open triradiate cartilage, diagnosed with EOS of idiopathic, congenital, neuromuscular, syndrome-related, or mesenchymal origin, and an expected significant spinal deformity (Cobb angle > 80°) at skeletal maturity. Skeletally mature patients, patients with severe deformity (Cobb angle > 100°) or rigidity (bends < 50°), and patients with prior spinal surgery or medically unmanaged systemic diseases were excluded. The intraoperative exclusion criterion was contraindication for the MLT.

The historical controls were, in principle, matched based on the same inclusion/exclusion criteria as the MLT patients, while taking into consideration that certain information was not available from the PSSG database. The inclusion criteria of the control group were patients 5–10 years old at time of index surgery, diagnosed with EOS of idiopathic, congenital, neuromuscular, syndrome-related, or mesenchymal origin, who received a DGR, RBGR, or MCGR bilateral implant system at index surgery with rod configuration of either spine-to-spine (DGR and MCGR, both upper and lower parts attached to spine) or rib-to-spine (RBGR, upper part attached to ribs and lower part to spine). Preoperative X-ray and Cobb angle were also required. Patients with severe deformity (Cobb angle > 100°) and patients with prior spinal surgery were excluded. The flexibility index of the control group was unknown in the PSSG database; therefore, the flexibility of the curve was not an eligibility criterion. From the pool of potential matches, controls were selected by applying three mandatory matching criteria: age (± 9 months), etiology of scoliosis, and magnitude of deformity of the primary curve (± 20° in Cobb angle). Secondary matching criteria were gender and spine length from T1–S1 (± 15%). When multiple matches fulfilling the criteria were available, the one meeting both mandatory and secondary criteria and with the least difference to the respective MLT patient was selected based on the following in decreasing priority order: smallest difference in age, Cobb angle, spinal length, and weight. If the same match was the best match for two MLT patients, the MLT patient without an alternative match was given priority.

### Surgical technique

Using a minimal-invasive technique, the MLT employs a four-rod construct with rigid proximal and distal fixation and newly developed apical pedicle screw-based spinal anchors that glide along two pairs of overlapping spinal rods [9]. The apical gliding anchors allow for maximal apical translation whilst permitting passive growth elongation of the spine. In the absence of suitable apical pedicles or inability to achieve apical capture then MLT should not be performed and is a contraindication to this growth guidance system. The surgical exposure for self-growth guidance is different than standard growing rods. The procedure begins with a midline skin incision over the targeted spinous process. A subperiosteal dissection is only performed at the proximal and distal ends to place fixed spinal anchors, ideally pedicle screws, as these segments (two or three) must fuse. For the intercalary gliding anchors at the apex, a trans-muscular dissection is used to minimize spontaneous fusion. The muscular fascia is detached lateral to the spinous process, preserving the Multifidus and Spinalis over the lamina while mobilizing the Longissimus and Iliocostalis laterally, similar to the Wiltse approach. This layer is later closed over the gliding implants and rods. Gliding pedicle anchors are inserted via this trans-muscular plane with minimal exposure of the transverse process to identify the starting point, ensuring the adjacent facet joint remains intact. Intraoperative imaging confirms accurate pedicle entry and insertion, as these screws endure significant forces. Two pairs of 5–6 mm titanium rods extend from the proximal to distal fixed anchors, overlapping at the intercalary segments. Gliding anchors maintain correction by keeping the rods parallel and engaged. As the spine grows, the fixed proximal rods shift away from the distal ones. Reduction manoeuvres consisting of apical translation, rod derotation are performed for deformity correction. The number of gliding anchors depends on the severity of the deformity. Location and number of gliding anchors are optimized for apical control and minimized to avoid spontaneous fusion.

## Outcome measures

The primary outcome measure was the number of reoperations of the spine in 3 years. Reoperations in MLT patients were classified as planned, unplanned, definitive, or unassigned at each follow-up. For control patients, details of reoperations were retrospectively extracted from the database according to the follow-up schedule and reoperations were classified as for MLT patients by the PSSG.

Secondary outcome measures were: radiographic outcomes (measured by a central, independent reviewer) including spinal lengths T1–T12 and T1–S1 (measured as the direct line from the midpoint of the chosen end plates [10]) and Cobb angles, standing heights (measured using a fixed wall mount), and quality of life (assessed using the validated, 24-item early-onset scoliosis questionnaire [EOSQ-24]) [11–13]. Adverse events (AEs, as defined by the ISO 14155 guideline [14]) were recorded only for the MLT group as anticipated or other AEs.

### Statistical analysis

The null hypothesis (H0) was: The number of reoperations within 3 years after initial surgery is 5 in both groups, assuming 1 reoperation every 6 months on average [15]. The alternative hypothesis (H1) was: The number of reoperations is lower in the MLT group.

The sample size of 68 was calculated [16] based on a 0.05 significance level, 80% power, mean number of 5 reoperations in the control group and 3 in the MLT group, and a 90% follow-up rate at 3 years. Of the 68 patients, 17 would be treated with MLT and 51 would be treated with control techniques (17 per control technique). Continuous variables were summarized using descriptive statistics. Statistical testing was performed using Chi-Square test for associations between categorical variables, Wilcoxon rank sum test for differences in ordinal, interval, or not normally distributed variables, and Fisher’s exact test for associations in contingency tables when expected count was < 5 in > 20% of the cells.

Time at risk in person-years was used to calculate reoperation rates in conditional Poisson regression. Generalized estimating equations were used to provide robust standard errors that account for repeated measures and varying time at risk. The rate ratio was determined by exponentiating the predicted log rate.

Kaplan Meier estimators were employed with log-rank tests [17] to analyze time to first reoperation using all available follow-up data, including those beyond 3 years.

The statistical analyses were performed using the software SAS version 9.4 M8 (SAS Institute Inc., Cary, NC, USA).

## Results

### Reoperations

**Fig. 3:**
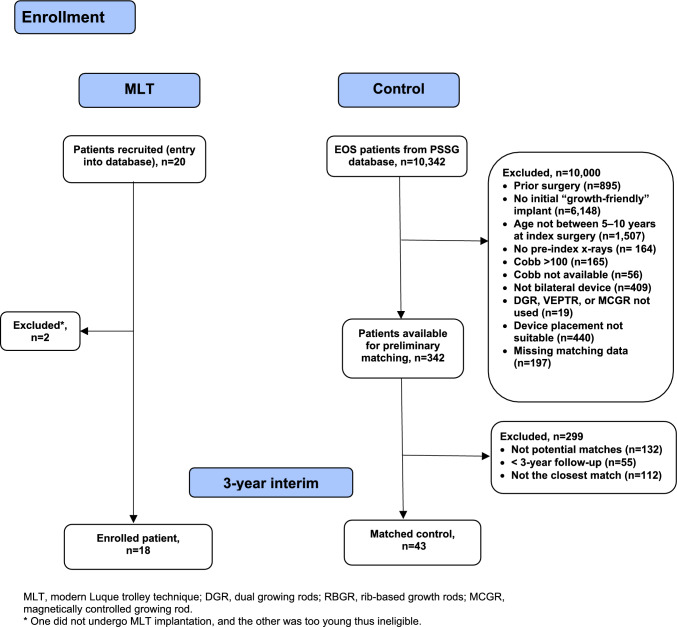
Patient flowchart

Eighteen patients were prospectively enrolled and treated with the MLT (MLT group); no patient dropped out in the 3-year follow-up period. The matching cohort included 43 patients: 16 treated with DGR, 9 with RBGR, and 18 with MCGR (Fig. 3). Thus, the planned 1:1 matching was fulfilled only for the MCGR. One MLT patient was found ineligible (Cobb angle > 100° or bends < 50°) in a retrospective review and was kept in the analysis, but a sensitivity analysis excluding this patient was performed. This report presents the primary outcome at 3 years, with the MLT cohort still in ongoing follow-up.

Baseline patient characteristics are shown in Table 1 and no differences were detected: The median (Q1; Q3) age of both MLT and control group was 7.0 years (6.0; 8.0) and the distribution of etiologies was similar, with idiopathic scoliosis (MLT group, n = 6, 33.3%; control group, n = 17, 39.5%) and neuromuscular scoliosis (MLT group, n = 6, 33.3%; control group, n = 13, 30.2%) as the predominant cases. The median Cobb angle of the MLT group was 72.0° (Q1; Q3, 62.0; 77.0) and the control group, 68.0° (58.0; 76.0).

**Table 1:** Baseline information

Variable	MLT group N = 18	Control group N = 43	Total N = 61	*P* value	Test statistic
Gender, n (%)	18	43	61	0.804*	χ^2^ = 0.06, *df* = 1
Female	9 (50.0)	23 (53.5)	32 (52.5)		
Male	9 (50.0)	20 (46.5)	29 (47.5)		
Age at surgery [years]				1.000**	Z = 0.00
n	18	43	61		
Median (Q1; Q3)	7.0 (6.0; 8.0)	7.0 (6.0; 8.0)	7.0 (6.0; 8.0)		
Weight [kg]				0.512**	Z = 0.66
n	18	43	61		
Median (Q1; Q3)	23.8 (18.7; 27.9)	21.0 (18.5; 26.4)	21.2 (18.6; 26.4)		
Standing height [cm]				0.142**	Z = 1.47
n	15	33	48		
Median (Q1; Q3)	121.0 (113.5; 128.0)	114.4 (110.5; 119.4)	115.6 (111.3; 125.8)		
Sitting height [cm]				0.757**	Z = − 0.31
n	14	12	26		
Median (Q1; Q3)	61.0 (56.0; 65.2)	60.5 (58.3; 62.0)	60.5 (57.2; 63.0)		
Early-onset scoliosis, n (%)					
Idiopathic	6 (33.3)	17 (39.5)	23 (37.7)	0.649*	χ^2^ = 0.21, * df* = 1
Neuromuscular	6 (33.3)	13 (30.2)	19 (31.1)	0.811*	χ^2^ = 0.06, * df* = 1
Syndrome-related	5 (27.8)	12 (27.9)	17 (27.9)	0.992*	χ^2^ = 0.00, * df* = 1
Congenital	1 (5.6)	1 (2.3)	2 (3.3)	0.507***	χ^2^ = 0.42, * df* = 1
Cobb angle [degree]				0.602**	Z = 0.52
n	18	43	61		
Median (Q1; Q3)	72.0 (62.0; 77.0)	68.0 (58.0; 76.0)	68.0 (59.0; 76.0)		
Spine length (T1–S1) [cm]				0.251**	Z = 1.15
n	18	43	61		
Median (Q1; Q3)	30.3 (28.0; 31.5)	28.7 (25.8; 31.1)	28.9 (26.2; 31.3)		
Spine length (T1–T12) [cm]				0.164**	Z = − 1.39
n	18	43	61		
Median (Q1; Q3)	17.3 (15.6; 18.0)	17.8 (16.2; 18.9)	17.5 (16.0; 18.6)		
Maximal sagittal kyphosis: Cobb angle [degree]				0.253**	Z = − 1.14
n	12	43	55		
Median (Q1; Q3)	42.0 (37.0; 48.5)	50.0 (35.0; 60.0)	47.0 (36.0; 58.0)		

*MLT* modern Luque trolley technique, *df* degree of freedom, *Q1; Q3* interquartile range

*Chi-square test; **Wilcoxon rank sum test; ***Fisher’s exact test. All tests used two-tailed p-values

There were no major differences in the growth-sparing construct between MLT and the control group with one exception that the RBGR had 50% of its cases with only 2 proximal anchors, compared to MLT, DRG and MCGR which had none. The percentages of cases with 4 proximal anchors in MLT, MCGR, DGR, and RBGR were respectively 89%, 56%, 44%, and 38%, while the percentages of cases with greater than 4 anchors respectively were 11%, 44%, 56%, and 12%. There were no differences in the number of segments spanned across the groups with a median of 14 levels spanned in both groups, with a range of 12–19 in MLT and a range of 11–16 in the control group. The most frequent upper end instrumented vertebra in MLT was T3, while in the control group it was T2. The most frequent lower end instrumented vertebra in MLT was L4 and in the control group it was L3 and L4.

Within 3 years after the surgery, only 1 (5.6%) MLT patient was reoperated due to proximal screw pullout, compared to 122 reoperations in 30 (69.8%) control patients with various causes for reoperation (Tables 2 and 6). Although no MCGR patients underwent planned reoperations, 4 unplanned revisions (in 2 patients), 1 definitive treatment, and 3 unassigned reoperations (in 3 patients) were recorded (Tables 2 and 3). No MLT patient had a definitive treatment or spontaneous spinal fusion.

**Table 2:** Number of patients reoperated within 3 years

	MLT (N = 18)	All controls (N = 43)	DGR (N = 16)	RBGR (N = 9)	MCGR (N = 18)
Patients with reoperations, n (%)	1 (5.6)	30 (69.8)	15 (93.8)	9 (100.0)	6 (33.3)
Planned revision^a^, n (%)	0	24 (55.8)	15 (93.8)	9 (100.0)	0 (0.0)
Unplanned revision^a^, n (%)	1 (5.6)	10 (23.3)	6 (37.5)	2 (22.2)	2 (11.1)
Definitive treatment^a^, n (%)	0	6 (14.0)	3 (18.8)	2 (22.2)	1 (5.6)
Unassigned^a^, n (%)	0 (0.0)	9 (20.9)	4 (25.0)	2 (22.2)	3 (16.7)
Number of reoperations per patient					
Median (Q1; Q3)	0.0 (0.0; 0.0)	4.0 (0.0; 5.0)	4.0 (4.0; 5.0)	5.0 (5.0; 6.0)	0.0 (0.0; 1.0)
Min; max	0.0; 1.0	0.0; 7.0	0.0; 7.0	4.0; 7.0	0.0; 3.0
Patients with known unplanned or definitive reoperations	1 (5.6)	14 (32.6)	7 (43.8)	4 (44.4)	3 (16.7)
Number of known unplanned or definitive reoperations per patient					
Median (Q1; Q3)	0.0 (0.0; 0.0)	0.0 (0.0; 1.0)	0.0 (0.0; 1.5)	0.0 (0.0; 1.0)	0.0 (0.0; 0.0)
Min; max	0.0; 1.0	0.0; 3.0	0.0; 3.0	0.0; 2.0	0.0; 3.0

All reoperations refer to reoperation of the spine

*MLT* modern Luque trolley technique, *DGR* dual growing rods, *RBGR* rib-based growing rods, *MCGR* magnetically controlled growing rod, *N* total number of patients, *n* number of patients with reoperations

^a^For the control group, the types of reoperations were retrospectively assigned

**Table 3:** Number of reoperations within 3 years

	MLT (N = 18)	All controls (N = 43)	DGR (N = 16)	RBGR (N = 9)	MCGR (N = 18)
Total number of reoperations	1	122	65	49	8
Planned revision^a^, n	0	90	48	42	0
Unplanned revision^a^, n	1	17	10	3	4
Definitive treatment^a^, n	0	6	3	2	1
Unassigned^a^, n	0	9	4	2	3

All reoperations refer to reoperation of the spine

*MLT* modern Luque trolley technique, *DGR* dual growing rods, *RBGR* rib-based growing rods, *MCGR* magnetically controlled growing rod, *N* total number of patients, *n* number of reoperations

^a^For the control group, the types of reoperations were retrospectively assigned

The conditional Poisson regression showed that the reoperation rates within 3 years were 0.06 (95% CI 0.01; 0.37) for the MLT group and 2.76 (95% CI 2.14; 3.57) for the control group, rate ratio 0.02 (95% CI 0; 0.12) (*P* < 0.001) (Table 4). A sensitivity analysis excluding the one ineligible patient resulted in the same rate ratio. All control techniques performed worse than the MLT, with the best-performing control technique, MCGR, having a reoperation rate ratio of 10.92 (95% CI 1.38; 86.6) (*P* = 0.024) comparing to the MLT. When the comparison was restricted to known unplanned or definitive reoperations, the rate ratio was 0.09 (95% CI 0.01; 0.62), *P* = 0.015, favoring the MLT (Table 4); individual comparisons showed better results for the MLT than DGR or RBGR, but not the MCGR (rate ratio, 6.83 [95% CI 0.71; 65.55], *P* = 0.096), Table 4.

**Table 4:** Reoperation rates and conditional Poisson regression analysis

Treatment group	Reoperation rate per 3 person-years (95% CI)	Rate Ratio (95% CI)	*P* value
All reoperations			
MLT vs control			
MLT	0.06 (0.01; 0.37)	0.02 (0; 0.12)	< 0.001
Control	2.76 (2.14; 3.57)	Reference	
Individual techniques vs MLT			
DGR	4 (3.28; 4.88)	80.21 (11.78; 546.11)	< 0.001
RBGR	5.25 (4.74; 5.81)	94.51 (13.92; 641.52)	< 0.001
MCGR	0.43 (0.19; 0.96)	10.92 (1.38; 86.6)	0.024
MLT	0.06 (0.01; 0.37)	Reference	
Known unplanned and definitive reoperations			
MLT vs control			
MLT	0.06 (0.01; 0.37)	0.09 (0.01; 0.62)	0.015
Control	0.53 (0.32; 0.88)	Reference	
Individual techniques vs MLT			
DGR	0.84 (0.44; 1.58)	17.26 (2.3; 129.74)	0.006
RBGR	0.55 (0.25; 1.25)	10.15 (1.25; 82.24)	0.030
MCGR	0.27 (0.08; 0.9)	6.83 (0.71; 65.55)	0.096
MLT	0.06 (0.01; 0.37)	Reference	

*MLT* modern Luque trolley technique, *DGR* dual growing rods, *RBGR* rib-based growing rods, *MCGR* magnetically controlled growing rod

**Fig. 4:**
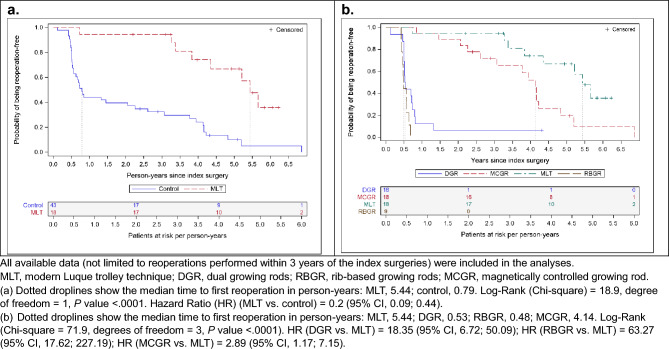
Kaplan Meier analysis of time to first reoperation by treatment group, all reoperations

**Fig. 5:**
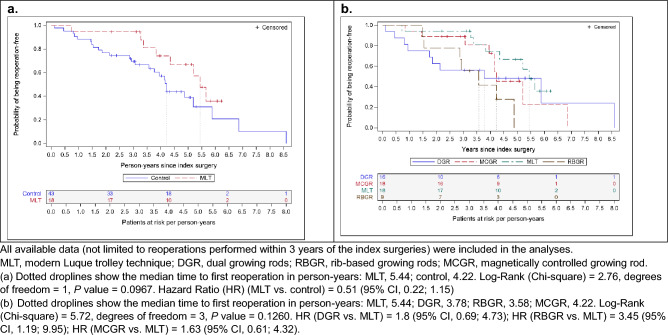
Kaplan Meier analysis of time to first reoperation by treatment group, known unplanned and definitive reoperations

Time to first reoperation was compared using all available data (mean [standard deviation, SD] follow-up time: MLT group, 4.8 [1.1] years; control group, 4.6 [1.5] years; DGR, 5.0 [1.7] years; RBGR, 4.6 [1.5] years; MCGR, 4.3 [1.3] years). The Kaplan Meier analysis calculated the time to first reoperation as MLT, 5.4 years and controls, 0.8 years, *P* < 0.0001, demonstrating a significant difference (Fig. 4a). Among the control techniques, MCGR had the longest time to first reoperation with 4.1 years (Fig. 4b). When the analysis was restricted to known unplanned or definitive reoperations, no difference was detected between the MLT and control group (Fig. 5a) or between MLT and individual control techniques (Fig. 5b).

### Secondary outcomes

**Fig. 6:**
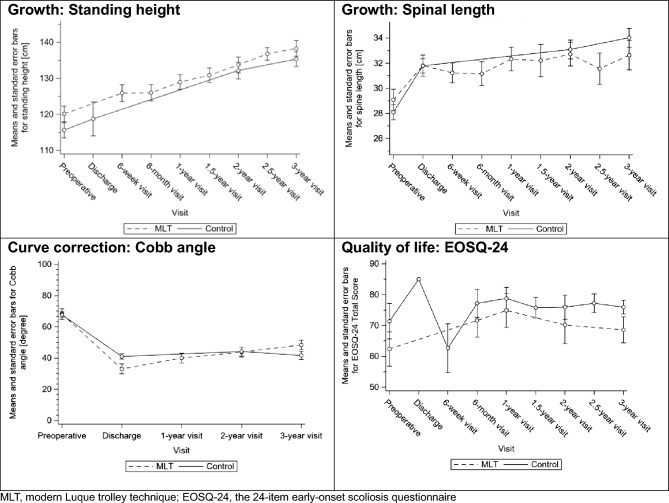
Comparison of standing heights, spinal lengths (T1–S1), Cobb angles, and quality of life

At discharge, MLT patients had a median T1–S1 spinal growth of 2.6 cm (Q1; Q3, 0.0; 4.9) and control patients, 3.4 cm (Q1; Q3, 2.2; 5.0) (*P* = 0.164), and at 3 years, these were 4.5 cm (Q1; Q3, 1.3; 6.8) and 4.8 cm (Q1; Q3, 3.0; 8.4), respectively (*P* = 0.156) (Table 5). Although the thoracic length (T1–T12) growth was similar at discharge (MLT, 1.5 cm [Q1; Q3, 0.0; 2.5]; control, 2.3 cm [Q1; Q3, 0.9; 3.5], *P* = 0.181), the control group had greater gain at 3 years (3.5 cm [Q1; Q3, 1.5; 5.6]) than the MLT group (2.3 cm [Q1; Q3, 0.8; 3.8]), *P* = 0.033 (Table 5). The growths in standing height were similar between the groups over time (Fig. 6).

**Table 5:** Change in secondary outcomes over time

Variable	Treatment group		*P* value	Test statistic
	MLT, N = 18	Control, N = 43		
Standing height [cm]				
Preoperative, n	15	33		
Median (Q1; Q3)	121.0 (113.5; 128.0)	114.4 (110.5; 119.4)	0.142^§^	Z = 1.47
3-year visit, n	13	38		
Median (Q1; Q3)	139.7 (131.0; 144.3)	135.0 (127.0; 143.7)	0.266^§^	Z = 1.11
3-year change from baseline, n	13	30		
Median (Q1; Q3)	17.8 (16.0; 19.2)	18.6 (16.5; 22.4)	0.420^§^	Z = − 0.81
3-year relative change as percentage of baseline value [%], n	13	30		
Median (Q1; Q3)	114.3 (112.9; 117.7)	115.9 (113.3; 120.0)	0.284^§^	Z = − 1.07
Total spine length (T1–S1) [cm]				
Preoperative, n	18	43		
Median (Q1; Q3)	30.3 (28.0; 31.5)	28.7 (25.8; 31.1)	0.251^§^	Z = 1.15
Discharge, n	16	42		
Median (Q1; Q3)	30.8 (29.5; 34.0)	32.1 (30.0; 34.0)	0.835^§^	Z = − 0.21
3-year visit, n	16	36		
Median (Q1; Q3)	32.5 (29.3; 35.3)	33.7 (30.6; 37.9)	0.293^§^	Z = − 1.05
Change from baseline at discharge, n	16	42		
Median (Q1; Q3)	2.6 (0.0; 4.9)	3.4 (2.2; 5.0)	0.164^§^	Z = − 1.39
Change from baseline at 3 years, n	16	36		
Median (Q1; Q3)	4.5 (1.3; 6.8)	4.8 (3.0; 8.4)	0.156^§^	Z = − 1.42
3-year relative change as percentage of baseline value [%], n	16	36		
Median (Q1; Q3)	116.0 (104.0; 121.6)	118.2 (110.6; 128.4)	0.194^§^	Z = − 1.30
Thoracic spine length (T1–T12) [cm]				
Preoperative, n	18	43		
Median (Q1; Q3)	17.3 (15.6; 18.0)	17.8 (16.2; 18.9)	0.164^§^	Z = − 1.39
Discharge, n	17	42		
Median (Q1; Q3)	18.0 (17.0; 19.0)	20.0 (18.3; 21.5)	0.044^§^	Z = − 2.02
3-year visit, n	16	37		
Median (Q1; Q3)	19.0 (16.8; 20.3)	21.5 (19.1; 23.4)	0.004^§^	Z = − 2.89
Change from baseline at discharge, n	17	42		
Median (Q1; Q3)	1.5 (0.0; 2.5)	2.3 (0.9; 3.5)	0.181^§^	Z = − 1.34
Change from baseline at 3 years, n	16	37		
Median (Q1; Q3)	2.3 (0.8; 3.8)	3.5 (1.5; 5.6)	0.033^§^	Z = − 2.13
3-year relative change as percentage of baseline value [%], n	16	37		
Median (Q1; Q3)	112.8 (104.2; 125.3)	121.3 (107.7; 130.1)	0.108^§^	Z = − 1.61
Cobb angle [degree]				
Preoperative, n	18	43		
Median (Q1; Q3)	72.0 (62.0; 77.0)	68.0 (58.0; 76.0)	0.602^§^	Z = 0.52
Discharge, n	17	43		
Median (Q1; Q3)	33.0 (24.0; 42.0)	39.0 (33.0; 45.0)	0.044^§^	Z = − 2.01
3-year visit, n	16	41		
Median (Q1; Q3)	52.0 (37.0; 55.0)	45.0 (28.0; 52.0)	0.106^§^	Z = 1.62
Change from baseline at discharge, n	17	43		
Median (Q1; Q3)	− 40.0 (− 44.0; − 30.0)	− 27.0 (− 35.0; − 19.0)	0.023^§^	Z = − 2.28
Change from baseline at 3 years, n	16	41		
Median (Q1; Q3)	− 21.0 (− 31.0; − 11.0)	− 22.0 (− 31.0; − 14.0)	0.613^§^	Z = 0.51
Correction at 3 years as percentage of initial correction [%], n	15	41		
Median (Q1; Q3)	− 48.8 (− 78.4; − 18.9)	− 16.7 (− 41.4; 34.0)	0.008^§^	Z = − 2.67
EOSQ-24 total score				
Preoperative, n	17	10		
Median (Q1; Q3)	61.2 (43.8; 85.4)	74.1 (69.3; 86.7)	0.380^§^	Z = 0.88
3-year visit, n	15	23		
Median (Q1; Q3)	70.0 (59.6; 83.8)	75.1 (68.0; 87.5)	0.170^§^	Z = − 1.37
3-year change from baseline, n	14	6		
Median (Q1; Q3)	2.7 (− 0.3; 17.9)	1.7 (− 5.6; 3.3)	0.433^§^	Z = − 0.78
3-year relative change as percentage of baseline value [%], n	14	6		
Median (Q1; Q3)	106.9 (99.2; 129.3)	102.3 (92.2; 103.8)	0.433^§^	Z = − 0.78

*MLT* modern Luque trolley technique, *EOSQ-24* the 24-item early-onset scoliosis questionnaire

^§^Wilcoxon rank sum test

At discharge, the MLT group had a better initial correction (median: − 40.0° [Q1; Q3, − 44.0; − 30.0]) than the control group (− 27.0° [Q1; Q3, − 35.0; − 19.0]), *P* = 0.023. At 3 years, however, the MLT group had greater loss of correction (− 48.8% [Q1; Q3, − 78.4; − 18.9)] than the control group (− 16.7% [Q1; Q3, − 41.4; 34.0]), *P* = 0.008, resulting in a similar amount of correction (MLT: − 21.0° [Q1; Q3, − 31.0; − 11.0]; control: − 22.0° [− 31.0; − 14.0], *P* = 0.613) (Table 5). The median Cobb angles at 3 years were MLT, 52.0° (Q1; Q3, 37.0; 55.0) and control, 45.0° (Q1; Q3, 28.0; 52.0) (*P* = 0.106).

No differences were observed in EOSQ-24 scores (Fig. 6 and Table 5).

### Safety analysis

Both anticipated and other AEs occurred within 3 years are reported in Table 6. In summary, 10 MLT patients had 11 AEs, including 2 occurrences of curve regression/recurrence of deformity and 1 implant loosening, leading to an estimated risk of about 11% for curve regression/recurrence of deformity, and about 6% for implant loosening (Table 6). Other AEs included 1 death (cause unknown after autopsy), 2 loss or reduced motor evoked potential during surgery, 1 broken femur, 2 pulmonary function problems, 1 change in curve morphology, and 1 change in shoulder balance.

**Table 6:** Adverse events occurring within 3 years of surgery, MLT group

Adverse events	Patient-level		Event-level
	N = 18		N = 11
	n^a^	%^b^ (95% CI^c^)	n
Any adverse event	10	55.6 (30.8; 78.5)	11
Spinal cord deficit	0	0.0 (0.0; 18.5)	0
Cauda equina deficit	0	0.0 (0.0; 18.5)	0
Nerve root deficit (motor deficit)	0	0.0 (0.0; 18.5)	0
Nerve root deficit (sensory deficit)	0	0.0 (0.0; 18.5)	0
Radiculopathy	0	0.0 (0.0; 18.5)	0
Spontaneous spinal fusion	0	0.0 (0.0; 18.5)	0
Growth arrest	0	0.0 (0.0; 18.5)	0
Curve regression/recurrence of deformity	2	11.1 (1.4; 34.7)	2
Outgrowth of construct	0	0.0 (0.0; 18.5)	0
Implant loosening	1	5.6 (0.1; 27.3)	1
Implant failure (bending/breakage of screws/rods, rupture of PEEK cables)	0	0.0 (0.0; 18.5)	0
Particle debris due to gliding motion of the rod	0	0.0 (0.0; 18.5)	0
Superficial wound infection	0	0.0 (0.0; 18.5)	0
Deep wound infection	0	0.0 (0.0; 18.5)	0
Other adverse events	8	44.4 (21.5; 69.2)	8

^a^Number of patients with at least one adverse event (AE). If a patient experienced multiple AEs under the same AE class, the patient was only counted once

^b^Estimated risk of developing at least one adverse event, calculated by dividing the number of patients experiencing at least one adverse event by the total number of patients

^c^Confidence intervals for percentages were calculated using the Clopper–Pearson method

## Discussion

Although EOS management using DGR was an initial standard of care, the need for repeated open lengthening is a serious drawback both psychosocially and medically [1, 6]. In theory, MCGR using closed distractions should eliminate repeated planned surgeries, however, retrospective reviews showed reoperation rates up to 45% at 50 months [7, 18]; newer techniques are therefore still being developed [1].

The original Luque trolley technique used two overlapping U-shaped rods and sublaminar wires for segmental fixation at proximal and distal ends, allowing for the rods to distract as the spine grows [3, 19]. Although initial results were promising, the technique was abandoned due to high rate of implant failure, spontaneous fusion, and poor curve control [4, 20]. The MLT addressed all three problems: Pedicle screws replaced the original sublaminar wires for fixation to the proximal and distal vertebra to reduce implant failure; non-locking, self-growing rods with gliding vehicles were developed to discourage spontaneous fusion; and minimal-invasive technique was employed to better preserve muscle and soft tissue to further reduce spontaneous fusion and complications. Additionally, the gliding vehicle, capturing the apex of the deformity, should help obtaining maximal apical translation and improve the initial deformity correction. In summary, the MLT should ideally provide self-guided spinal growth with reduced implant failure, maintain curve correction, and avoid repeating surgeries.

The current MLT series recorded one unplanned reoperation for implant failure and no spontaneous fusion within 3 years, in sharp contrast to the control group, where a sizeable number were recorded, and to the original Luque trolley where a 100% revision rate and a 100% autofusion rate at an average follow-up of 3 years were reported [4]. Although the current study did not capture the implant failure rate or autofusion from the control patients, the MLT performed better than data published for MCGR: 7 rod breakages in 6 patients and other implant-related complications in 7 patients in a study of 54 patients (mean follow-up, 19.4 months) [6], 15/47 patients (32%) with implant-related complications in another (mean follow-up, 50 months) [7], and 26% of 161 patients with implant-related complications needing revision in a third study [21]. Although MCGRs should be associated with less autofusion, multiple cases of autofusion have been documented [21–24]. The frequency of autofusion for MCGR is not well established [2] and therefore a comparison between the MLT and MCGR is not feasible. Finally, since MCGR is continuously being improved, it is possible that the high complication rates found in the literature reflected the performance of older generations of MCGR devices or surgical techniques.

The current result not only supports the alternative hypothesis that there would be fewer reoperations with MLT than control techniques, but MLT treatment also had a lower reoperation rate than published data in general. Although this is a short-term result of a relatively small series, it is very encouraging.

### Spinal growth and curve correction

In the current study, the T1–S1 spinal growth was similar between the MLT and control group at 3 years, but T1–T12 growth was smaller in the MLT group. Comparing spinal growths is not straightforward because the method of measurement and reporting are not standardized: some reported total growth (from preop to final follow-up), some reported follow-up growth (after initial surgery to final follow-up), and some reported true growth (after initial surgery to before final fusion) [10]. Our current report of total T1–S1 growth of 4.5 cm (Q1; Q3, 1.3; 6.8) and total T1–T12 growth of 2.3 cm (Q1; Q3, 0.8; 3.8) at 3 years in MLT patients is on the low end compare to the 1.4–3.4 cm per year reported in a systematic review of all growth-friendly systems [10]. However, in this systematic review, the reported values included studies reporting both growth with and without final fusion. Since none of our MLT patients had the final fusion, less growth should be expected.

In both MLT and control group, curve correction happened primarily at discharge. The loss of postoperative correction, however, was greater in the MLT group; the final Cobb angles at 3 years were similar in the two groups. Compared to the literature performance of MCGR, the amount of curve correction is similar to those reported by Lebel et al. (correct from 69.6° at preop to 52.8° at 50-month follow-up) [7] but less than Abdelaal et al. (from 70° at preop to 39° at 4.1-year follow-up) [18]. The large amount of loss in the MLT group is disappointing and longer-term follow-up is crucial to see if this trend continues.

Quality of life scores fluctuated over time in both groups and were not consistently available for the control patients. Additionally, the sample size calculation was not done to address this outcome, so the current result should not be taken as conclusive.

## Limitations and strengths

The main limitation is that although the data for the MLT group was prospectively collected, a historical cohort was used as the control. Not only data were collected differently, changes and improvements in devices or surgical techniques that influenced the results were not captured. Another limitation is the short follow-up period of 3 years. In addition, the envisioned 1:1 match for all control techniques was not achieved; this decreased the power. Nevertheless, the large difference in the reoperation rates observed between the MLT and the control group cannot be ignored—the significant difference in the reoperation rates shown in the conditional Poisson regression is expected to remain had the planned number of controls been reached. Lastly, a limitation of the study was we did not have the flexibility index of the control group, and this may have influenced the revision rate. However, it is unlikely as the initial correction percentage of the Cobb for the control group and the MLT were statistically different but clinically similar.

Compared with the current literature that consists mainly of retrospective reviews, the strength of the current study is the prospective collection of outcomes and comprehensive recording of adverse events after MLT treatment.

## Conclusion

Patients treated with the MLT had a greatly reduced reoperation rate than control patients within 3 years of surgery and low risk of implant failure. The thoracic spinal growth was less than the control group, but the total spinal growth and amount of curve correction were similar.

## Data Availability

The data for this study is not open access. Therefore a data availability statement is not applicable.

## References

[1] Studer D, Hasler CC (2024) Diagnostic and therapeutic strategies in early onset scoliosis: a current concept review. J Child Orthop 18(2):113–123. 10.1177/18632521241228141 38567043 10.1177/18632521241228141 PMC10984154

[2] Hatem A, Elmorshidy EM, Elkot A, Hassan KM, El-Sharkawi M (2024) Autofusion in growing rod surgery for early onset scoliosis; what do we know so far? SICOT J 10:15. 10.1051/sicotj/2024011 38687150 10.1051/sicotj/2024011 PMC11060050

[3] Luque ER, Cardoso A (1977) Treatment of scoliosis without arthrodesis or external support, preliminary report. Orthop Trans 1977(1):37–38

[4] Mardjetko SM, Hammerberg KW, Lubicky JP, Fister JS (1992) The Luque trolley revisited. Review of nine cases requiring revision. Spine 17(5):582–589. 10.1097/00007632-199205000-00018 1621159 10.1097/00007632-199205000-00018

[5] Klyce W, Mitchell SL, Pawelek J, Skaggs DL, Sanders JO, Shah SA et al (2020) Characterizing use of growth-friendly implants for early-onset scoliosis: a 10-year update. J Pediatr Orthop 40(8):e740–e746. 10.1097/BPO.0000000000001594 32467421 10.1097/BPO.0000000000001594

[6] Choi E, Yaszay B, Mundis G, Hosseini P, Pawelek J, Alanay A et al (2017) Implant complications after magnetically controlled growing rods for early onset scoliosis: a multicenter retrospective review. J Pediatr Orthop 37(8):e588–e592. 10.1097/BPO.0000000000000803 27328123 10.1097/BPO.0000000000000803

[7] Lebel DE, Rocos B, Helenius I, Sigal A, Struder D, Yazici M et al (2021) Magnetically controlled growing rods graduation: deformity control with high complication rate. Spine 46(20):E1105–E1112. 10.1097/BRS.0000000000004044 34559751 10.1097/BRS.0000000000004044

[8] Ouellet J (2011) Surgical technique: modern Luque trolley, a self-growing rod technique. Clin Orthop Relat Res 469(5):1356–1367. 10.1007/s11999-011-1783-4 21274761 10.1007/s11999-011-1783-4 PMC3069294

[9] Navarro-Ramirez R, Rabau O, Teles A, Ge S, Shebreen AB, Saran N, Ouellet J (2020) A novel growing rod technique to treat early-onset scoliosis (EOS): a step-by-step 2D surgical video. Neurosurg Focus Video 2(1):V9. 10.3171/2020.1.FocusVid.19683 36284693 10.3171/2020.1.FocusVid.19683 PMC9521204

[10] Wijdicks SPJ, Tromp IN, Yazici M, Kempen DHR, Castelein RM, Kruyt MC (2019) A comparison of growth among growth-friendly systems for scoliosis: a systematic review. Spine J 19(5):789–799. 10.1016/j.spinee.2018.08.017 30290228 10.1016/j.spinee.2018.08.017

[11] Corona J, Matsumoto H, Roye DP, Vitale MG (2011) Measuring quality of life in children with early onset scoliosis: development and initial validation of the early onset scoliosis questionnaire. J Pediatr Orthop 31(2):180–185. 10.1097/BPO.0b013e3182093f9f 21307713 10.1097/BPO.0b013e3182093f9f

[12] Matsumoto H, Williams B, Park HY, Yoshimachi JY, Roye BD, Roye DP Jr et al (2018) The final 24-Item Early Onset Scoliosis Questionnaires (EOSQ-24): validity, reliability and responsiveness. J Pediatr Orthop 38(3):144–151. 10.1097/BPO.0000000000000799 27299779 10.1097/BPO.0000000000000799 PMC5562528

[13] Vitale MG, Corona J, Matsumoto H, Avendano J, Pinder D, Miller DJ, Roye DP (2010) Development and initial validation of a disease specific outcome measure for early onset scoliosis. Stud Health Technol Inform 158:172–176 20543419

[14] ISO Technical Committee ISO/TC 194 (2020) ISO 14155:2020 Clinical investigation of medical devices for human subjects—good clinical practice, 3rd edn. International Organization for Standardization, Geneva

[15] Sankar WN, Skaggs DL, Yazici M, Johnston CE 2nd, Shah SA, Javidan P et al (2011) Lengthening of dual growing rods and the law of diminishing returns. Spine 36(10):806–809. 10.1097/BRS.0b013e318214d78f 21336236 10.1097/BRS.0b013e318214d78f

[16] van Belle G. Statistical Rules of Thumb (Wiley Series in Probability and Statistics). 2 ed: Wiley; 2008 2008

[17] Kaplan EL, Meier P (1958) Nonparametric estimation from incomplete observations. J Am Stat Assoc 53(282):457–481. 10.1080/01621459.1958.10501452

[18] Abdelaal A, Munigangaiah S, Trivedi J, Davidson N (2020) Magnetically controlled growing rods in the treatment of early onset scoliosis: a single centre experience of 44 patients with mean follow-up of 4.1 years. Bone Jt Open 1(7):405–414. 10.1302/2633-1462.17.BJO-2020-0099.R1 33215131 10.1302/2633-1462.17.BJO-2020-0099.R1 PMC7659683

[19] Luque ER (1982) Paralytic scoliosis in growing children. Clin Orthop Relat Res 163:202–209 7067255

[20] Pratt RK, Webb JK, Burwell RG, Cummings SL (1999) Luque trolley and convex epiphysiodesis in the management of infantile and juvenile idiopathic scoliosis. Spine 24(15):1538–1547. 10.1097/00007632-199908010-00007 10457573 10.1097/00007632-199908010-00007

[21] Glowka P, Grabala P, Gupta MC, Pereira DE, Latalski M, Danielewicz A et al (2024) Complications and health-related quality of life in children with various etiologies of early-onset scoliosis treated with magnetically controlled growing rods-a multicenter study. J Clin Med 13(14):4068. 10.3390/jcm13144068 39064107 10.3390/jcm13144068 PMC11277853

[22] Cheung JPY, Sze KY, Cheung KMC, Zhang T (2021) The first magnetically controlled growing rod (MCGR) in the world—lessons learned and how the identified complications helped to develop the implant in the past decade: case report. BMC Musculoskelet Disord 22(1):319. 10.1186/s12891-021-04181-0 33794851 10.1186/s12891-021-04181-0 PMC8015050

[23] Green AH, Brzezinski A, Ishmael T, Adolfsen S, Bowe JA (2021) Premature spinal fusion after insertion of magnetically controlled growing rods for treatment of early-onset scoliosis: illustrative case. J Neurosurg Case Lessons 2(17):Case21446. 10.3171/case21446 36060899 10.3171/CASE21446 PMC9435560

[24] Yang MJ, Rompala A, Samuel SP, Samdani A, Pahys J, Hwang S (2023) Autofusion with magnetically controlled growing rods: a case report. Cureus 15(3):e36638. 10.7759/cureus.36638 37155436 10.7759/cureus.36638 PMC10122916

